# Exploration and Practice of “Internet + Maker Education” University Innovative Entrepreneurship Education Model From the Perspective of Positive Psychology

**DOI:** 10.3389/fpsyg.2020.00891

**Published:** 2020-06-08

**Authors:** Xiaomeng Sun

**Affiliations:** ^1^School of Economics, Zhejiang Gongshang University, Hangzhou, China; ^2^Graduate School, University of Perpetual Help System DALTA, Las Piñas, Philippines

**Keywords:** positive psychology, the internet, maker education, innovative entrepreneurship, education model

## Abstract

This thesis aims to analyze the importance of psychological quality in the entrepreneurial education for undergraduates in the case of positive psychology, to explore an educational model that combines the Internet information technology with maker education, and to culture the innovative entrepreneurial capabilities of undergraduates. First, the theories of positive psychology were reviewed worldwide, and the current cultivation of entrepreneurial psychological quality for undergraduates was summarized. Second, undergraduates were taken as the survey objects. The questionnaires were distributed both online and offline to investigate the innovative entrepreneurship education (the IE education) in three different universities in Zhejiang Province, China. The current development of IE education in universities was summarized. Finally, according to the current educational status in universities, combining the Internet and maker education, the implementation strategies suitable for IE education in universities were proposed. According to the results of questionnaire survey, the proportion of college students who chose entrepreneurship was 6.1%. The innovation and entrepreneurship of contemporary college students was not optimistic, which was mainly related to the lack of systematic planning and teachers in China’s current innovation and entrepreneurship education. Through the integration of Internet information technology and maker education, this study constructed a more systematic implementation scheme of innovation and entrepreneurship education in colleges and universities. It could meet the needs of students of different majors and grades. Meanwhile, teachers should give full play to the Students’ subjective initiative and emphasize the student-centered learning style, thereby promoting the cultivation of students’ innovative thinking. The IE education model of “Internet + Maker Education” in colleges can reform traditional college teaching methods and promote the cultivation of entrepreneurship, innovative thinking, and creative abilities, and the model can be used as a new idea and method for the development of IE education in universities.

## Introduction

Since the twenty-first century, China has new requirements for the development of ideology on a certain economic basis. In addition, the innovative entrepreneurship education (the IE education) in universities is an educational model that is compatible with socioeconomic development and education reform, which should reflect the integration, innovation, and application of knowledge (Rodney and Jacob, 2017). Therefore, the practice of this new education model is also an essential task for undergraduates. When referring to the reform of higher education in the *Report to the Eighteenth National Congress of the Communist Party of China*, it was proposed that “We should turn China into a country with a large pool of competent professionals, and give more support to the training of innovative and entrepreneurial personnel” (Alicia and Christopher, 2017; Jiang et al., 2017; Svenna et al., 2019). The Chinese leader also pointed out in the *Report at the 19th National Congress of the Communist Party of China* that “Innovation is the primary driving force behind development; it is the strategic underpinning for building a modernized economy.” Meanwhile, the Chinese leader also emphasized the importance of innovation and development in the new era (Jesse et al., 2018). Therefore, if China wants to adhere to the innovation-driven development strategy, it is necessary to follow the trend of the times and put forward new requirements for IE education of undergraduates.

With the continuous popularization of applying Internet technology in various fields, the advantages of the Internet and science and technology have also caused drastic changes in the daily lives of people, which even have a subtle influence on the thinking modes of people (Deborah et al., 2018). Given the current information age, the educational form of Chinese universities has also changed, and the cultivation of innovative talents is an inevitable trend in educational reform. Ge has also done some research on college student innovation in Chinese enterprises (Ge, 2020). Generally, undergraduates in China lack the innovative thinking and creative ability. Meanwhile, the knowledge learned in schools cannot be flexibly applied to practice. As a result, the problems of difficult employment and job selection for undergraduates have become increasingly apparent (Uche et al., 2017). In addition, Internet-based learning is an effective way to promote education informationization. It not only provides learning resources for maker education but also promotes communication between learners effectively. Moreover, it also promotes the process of change in teaching and learning methods, thereby continuously cultivating excellent innovative talents (Emanuela and Panagiotis, 2017; Lin and Wei, 2017). With the development of Internet information technology, the limitations of simple offline physical environment and communication are unable to meet the needs of university students’ maker learning, making it difficult to fully realize true cross-campus and interdisciplinary learning. For makers in colleges and universities, it is necessary to build a virtual online learning space to support the maker education. In the context of “mass entrepreneurship and innovation,” the implementation of maker education not only improves the innovative ability of university students but also creates more job opportunities and eases the employment pressure of university students.

At present, the college IE education model in China has just been initiated, which needs to be explored and tried continuously. On the one hand, it is necessary to propose effective reform strategies for the problems in the current IE education model. On the other hand, it is necessary to consider the effect of the psychological quality of undergraduates on the cultivation of their innovative thinking in the entrepreneurial process (Evans et al., 2018). The positive psychology of undergraduates is directly related to their academic development and healthy growth. Entrepreneurial psychological quality is a comprehensive psychological quality formed and developed by individuals under the influence of environment and education. It is a stable and comprehensive personality psychological characteristic that regulates the psychology and behavior of an individual in the practice of entrepreneurship. Therefore, focusing on the cultivation of positive psychology of undergraduates is not only beneficial to the integrity of the personality but also a vital prerequisite for cultivating innovative talents with comprehensive development.

In summary, this study analyzes the importance of psychological quality in the entrepreneurial process of undergraduates in the case of positive psychology, explores an educational model that combines the Internet information technology with maker education, and cultivates the innovative and entrepreneurial capabilities of undergraduates. While improving university students’ participation in maker education, maker education can also be combined with online virtual spaces for students to learn and create works, as well as better cultivating university students’ creative thinking and innovative entrepreneurship. In view of the current situation of university education, combining Internet with maker education, this study puts forward a set of strategies suitable for improving university innovation and entrepreneurship education mode, as a new thinking and method for the development of university innovation and entrepreneurship education.

## Materials and Methods

### Cultivation Contents of Efficient Student Innovative Entrepreneurship in the Case of Positive Psychology

Positive psychology advocates studying the positive psychological qualities of people. Meanwhile, positive psychological qualities refer to the fusion of a series of positive qualities, such as cognition, emotion, and behavior. Chinese scholars have studied the structure of positive psychological qualities for undergraduates. Finally, they have divided the dimensions of positive psychological qualities for undergraduates into six parts, i.e., cognition, interpersonal connection, emotion, justice, temperance, and transcendence (Daniel et al., 2018). Among these, “cognition” is the process by which people recognize outside things through the sensory organs. In this dimension, the creativity and curiosity of undergraduates can be cultivated. “Interpersonal connection” refers to the relationship formed between people in the process of social interaction. The positive psychological qualities of undergraduates in this dimension include sincerity, courage, and enthusiasm. The positive psychological qualities of the “emotion” dimension include feeling love, love and friendliness, and social cognition. The “justice” dimension is a positive psychological characteristic that involves the group domain and can help groups achieve their goals effectively and harmoniously. “Temperance” is to keep the original goodness and beauty of the dimension. Undergraduates should be tolerant and self-tempered to achieve long-term goals. “Transcendence” is the highest level of positive psychological characteristics of undergraduates, which aims to surpass reality and pursue a higher spiritual realm (Dirk, 2017; Tavassoli and Trippl, 2017; Bell et al., 2018).

In the case of positive psychology, the cultivation of innovative and entrepreneurial ability of contemporary undergraduates needs to start from four aspects, i.e., consciousness, will, ability, and personality. (1) The entrepreneurial consciousness and the entrepreneurial activities that undergraduates dominate and generate have a significant effect on the development of individuals and nations. Consciousness is the guide to action. Entrepreneurship consciousness reflects the social nature of entrepreneurial quality and governs the attitude and behavior of entrepreneurs toward entrepreneurial activities, which is an essential part of entrepreneurial quality. (2) Will refers to a long-term and stable psychological state that people generate to achieve their goals. The entrepreneurial will of undergraduates refers to the quality of entrepreneurs facing entrepreneurial risks and obstacles by adjusting their mentality. (3) The cultivation of entrepreneurial ability has a great relationship with the knowledge reserve, which mainly refers to the ability to transform knowledge and skills into practical applications, including decision-making ability, innovation ability, and management ability. (4) The entrepreneurial personality requires that the entrepreneurial individuals should have the consciousness of continuously seeking self-breakthrough and boldly trying to improve their competitiveness, thereby realizing their values (Ebru and Pinar, 2018).

### Contents and Implementation Strategies of Maker Education

In a broad sense, a maker refers to a person who turns a creative idea into reality; in a narrow sense, a maker refers to a person who realizes a creative idea through open-source software and hardware. At present, maker education can be regarded as a series of comprehensive courses, which can be skills training or innovative practical courses to students’ creative ability. Others believe that maker education is a maker activity, which refers to a learning activity where students will realize their creativity in the maker space. Maker education is considered as a new path for future education reform and innovation due to its distinctive innovation characteristics.

The teaching modes of traditional colleges and universities mainly focus on the dissemination of knowledge. Most of the teaching modes are collective teaching, whose emphasis on student learning is not much. The maker education to be carried out mainly focuses on the learning initiative of students and the interests given to students, which is project-based learning. At present, most colleges and universities still focus on traditional classroom teaching. Teachers impart knowledge, whereas students sit and listen passively without adequate practices (Newton et al., 2018). Under this teaching mode, students are in a passive and fixed state, which not only is unhelpful for the development of their personality but also lacks the interaction between teachers and students. Therefore, it is also not conducive to the cultivation of students’ autonomous learning ability.

By contrast, maker education advocates project-based learning. While students choose projects based on their interests, teachers provide appropriate guidance during the learning process. Teachers can also participate in making products with students. Maker education encourages students to cooperate and exchange across regions, disciplines, and subjects; enrich their knowledge; and expose themselves to more knowledge and technology, which helps students enhance their friendships in the process of thought collision. Maker education allows students to use current computers, artificial intelligence technologies, and 3D printing technology to achieve results through interdisciplinary teamwork (Seyranian et al., 2018). Through the process of discovering and solving problems, students can cultivate and improve their abilities in teamwork, innovation, and practice. Maker education just makes up for the shortcomings of the simple teaching goal of colleges and universities, provides students with opportunities for practice, gives full play to the creative thinking of students, exercises their ability to operate, and also complies with the national policy requirements for innovative and talent training in colleges and universities.

### IE Education Model Based on “Internet + Maker Education”

At present, maker education in Chinese universities is mainly centered on offline maker space; however, this form of education is limited by time and region (Michael, 2017). The virtual network learning environment created by the Internet, as an open place of activity, is not constrained by time and space. In addition, after the virtual scenes are constructed, undergraduates can have a real experience on the network platform (Kim et al., 2017). Currently, since the educational information is gradually being surrounded by Internet technology, the effective application of online space is more meaningful for the development of maker education. This study compares online and offline forms of maker education. The advantages of online maker education become evident. The specific comparison results are shown in Table 1.

**TABLE 1:** The comparison of education models between online and offline maker education.

**Space**	**Function**	**The main form of activity**
Online space	Learning	Choose courses to study online and submit assignments online; upload and download resources
	Communication	Communicate and discuss with classmates or teachers synchronously or asynchronously; posts, online comments, etc.
	Operation	Design, model, and combine physical tools or instrumentation operations online
	Show	Network platform display; for all users of the platform to browse
	Teachers’ guidance	n-school or cross-school teachers, entrepreneurs’ online guidance, answer questions
Offline space	Learning	Use multimedia in physical classrooms or laboratories
	Communication	Face-to-face communication and discussion between classmates or teachers
	Operation	Use 3D printer, laser cutting machine, and other processing tools to operate
	Show	Physical space exhibition area for teachers and undergraduates to visit
	Teachers’ guidance	On-campus teachers or external experts to the school guidance

**FIGURE 1:**
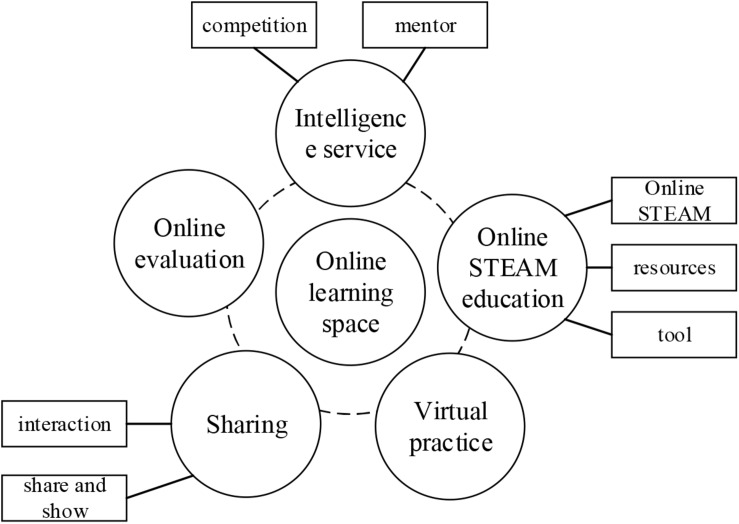
The construction diagram for the Internet platform of maker education.

In the current “Internet+” era, the online maker education form for undergraduates is gradually popularized and promoted. Meanwhile, the seamless development of offline and online education can promote the comprehensive development of maker education (Van der Helm et al., 2019). Based on the thinking of “Internet + Maker Education” and the current situation of maker education in universities, this study proposed the construction structure of maker education on the Internet platform based on the function of learning space, as shown in Figure 1.

**FIGURE 2:**
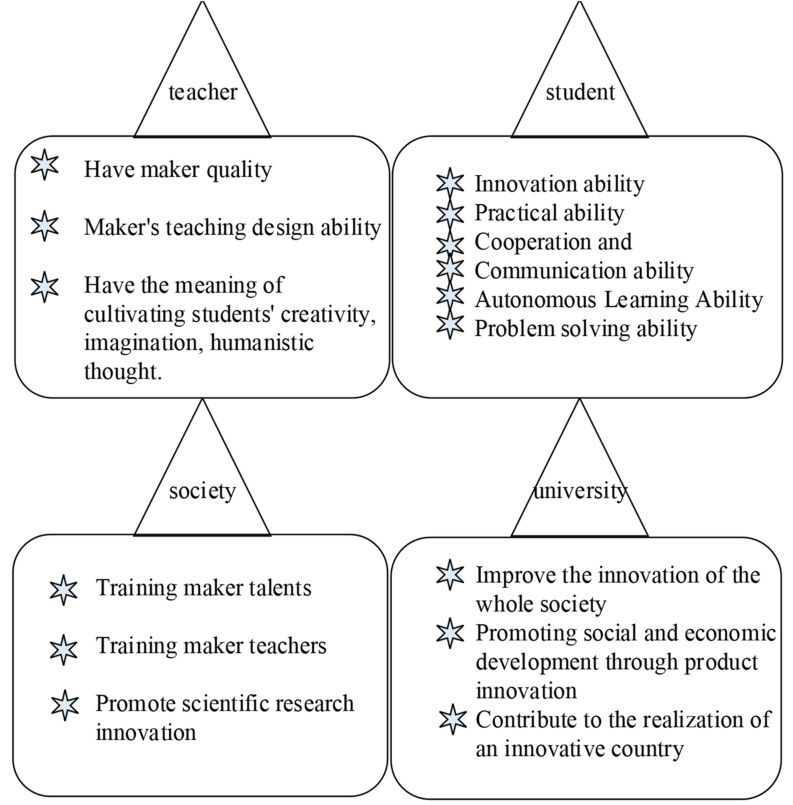
Goal positioning requirements of the four major bodies of maker education in universities.

From a macro perspective, the implementation of maker education mainly focuses on undergraduates, teachers, schools, and society. The specific requirements for the positioning of these four major goals are shown in Figure 2. Teachers are mainly responsible for cultivating undergraduates. The main purpose is to train undergraduates to become makers. Therefore, the teachers themselves must be makers of makers. In the maker education model, undergraduates mainly develop toward maker talents. Therefore, the target positioning of undergraduates mainly depends on the general process of maker activities. Universities are the major environments for cultivating maker talents (Van der Helm et al., 2019). In such an environment, teaching and scientific research work are completed. The society should provide the necessary support to the implementation of maker education, and the results of maker education implemented by universities will also affect society (Anne et al., 2017; Chien and Chu, 2017).

### Empirical Analysis

Although China has always valued the IE education of undergraduates, the position of IE education has always been at a disadvantage in the actual education process of universities. According to relevant documents, this study designed a set of questionnaires based on the theoretical results of research on IE education worldwide, expecting to have a preliminary understanding of the current situation of innovative entrepreneurship of undergraduates in China.

1. Survey objects: Undergraduates from three universities in Zhejiang Province were selected as survey objects. A total of 380 questionnaires were issued, of which 362 were retrieved and 18 invalid questionnaires were removed. Therefore, there were 344 valid questionnaires. The valid rate was 90.53%.
2. The major content of the questionnaire: The attitude of undergraduates in innovative entrepreneurship (self and family), the actual situation of universities in implementing IE education, and the level of entrepreneurial ability of undergraduates (basic ability, core ability, and social ability). The actual situation of implementing IE education in the three universities was evaluated from the aspects of teacher input, curriculum setting and resource provision. According to the Likert Scale, 1 to 5 points respectively represented 5 levels of extremely disagree, disagree, general, agree, and extremely agree. The ability scale in *Research on Existing Problems and Countermeasures of Innovative entrepreneurship* was selected to investigate the level of entrepreneurial ability, and the Likert 5-leveled Scale was used for scoring (Wouters et al., 2018).
3. Statistical methods of survey results: This study used Excel and SPSS 26.0 software to statistically analyze the survey results. The test statistic of the analysis of variance adopted the *F*-value test. The presence or absence of significant differences was represented by the *P*-value, and *P* < 0.05 indicates that the difference is statistically significant.

## Results

### Status and Analysis of IE Education for Undergraduates

**FIGURE 3:**
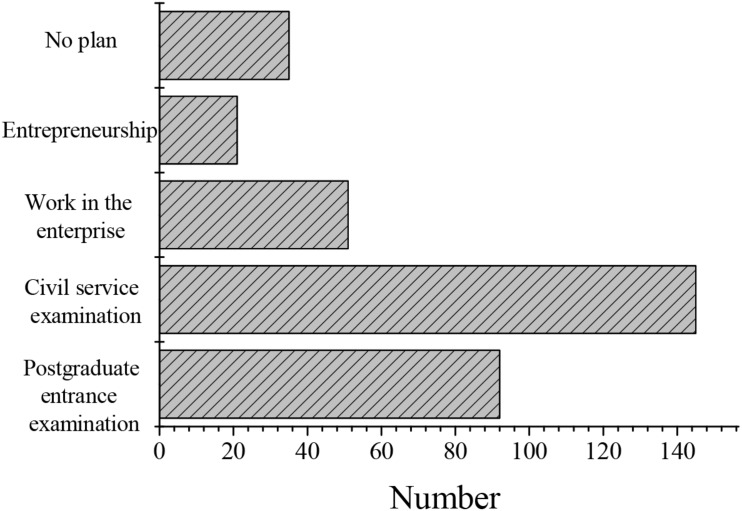
Attitudes and intentions of undergraduates in innovative entrepreneurship.

1. The attitudes of undergraduates toward innovative entrepreneurship: The choices of career after graduation of undergraduates were surveyed through a questionnaire. The selected content included five aspects, i.e., postgraduate entrance examination, civil service examination, work in the enterprise, entrepreneurship, and no plan. Statistics on the final results (Table 2 and Figure 3) could indirectly reflect the attitude of undergraduates to innovative entrepreneurship. As shown in Table 2, the proportion of undergraduates who chose to take the civil service examination for public institutions was relatively large (42.2%), while the proportion of undergraduates who started business was the least (6.1%). It reflects that the form of innovative entrepreneurship of contemporary undergraduates is not optimistic.

**TABLE 2:** Survey results of career selections of undergraduates after graduation.

**Direction**	**Postgraduate entrance examination**	**Civil service examination**	**Work in the enterprise**	**Entrepreneurship**	**No plan**
Number	92	145	51	21	35

2. The attitudes of families toward the innovative entrepreneurship of undergraduates: Since the attitudes of parents are a key influencing factor for the innovative entrepreneurship of undergraduates, a general understanding of parents’ attitudes was obtained through student feedback in the survey. Except for 88 undergraduates who had unclear attitudes toward their families, 148 (43.0%) undergraduates reported that their families were opposed to their entrepreneurship. In addition, 80 (23.3%) student parents were on the sidelines of student entrepreneurship and remained neutral attitude, while only 28 (8.1%) student parents supported the idea of entrepreneurship of their children and were willing to help. Therefore, the current situation of innovative entrepreneurship of undergraduates is not optimistic. A large part of it is affected by family factors, which is why undergraduates lack entrepreneurial motivation.
3. The actual situation of implementing IE education in three universities: For the 10 questions designed in this study, the specific survey results are shown in Table 3. As shown in Table 3, there are significant differences in the specific practice of IE education in the three universities in Zhejiang Province investigated in this study. Through the summary, it is found that the strength and resource support of teachers will have a significant effect on the establishment of undergraduates’ concepts of innovative entrepreneurship. Teachers with their entrepreneurial experience background and strong teaching ability can meet the problems of undergraduates in entrepreneurship. Also, in colleges that can provide sufficient resources, such as financial support and expert lectures, undergraduates will have more ideas about innovative entrepreneurship.

**TABLE 3:** The current situations of IE education in universities and colleges.

**Problem**	**df**	***F***	***P*-value**
Have a sense of harvest in learning innovative entrepreneurship courses	2	7.044	0.001
Innovative entrepreneurship is permeated in the setting of professional courses	2	10.047	0.001
The school will often invite successful entrepreneurs to give lectures	2	12.386	0.000
School IE education mainly focuses on teachers’ theoretical explanation	2	5.872	0.000
The school has an innovative entrepreneurship association	2	4.287	0.000
Teachers of innovative entrepreneurship course have rich experience in independent entrepreneurship	2	14.289	0.000
The school provides sufficient innovative entrepreneurship support funds	2	8.355	0.000
Teachers can solve problems in time in the process of innovative entrepreneurship	2	13.294	0.000
The innovative entrepreneurship activities held by the school are highly related to the theoretical courses	2	9.048	0.001
The school practice platform meets the needs of undergraduates’ innovative entrepreneurship	2	11.324	0.000

**FIGURE 4:**
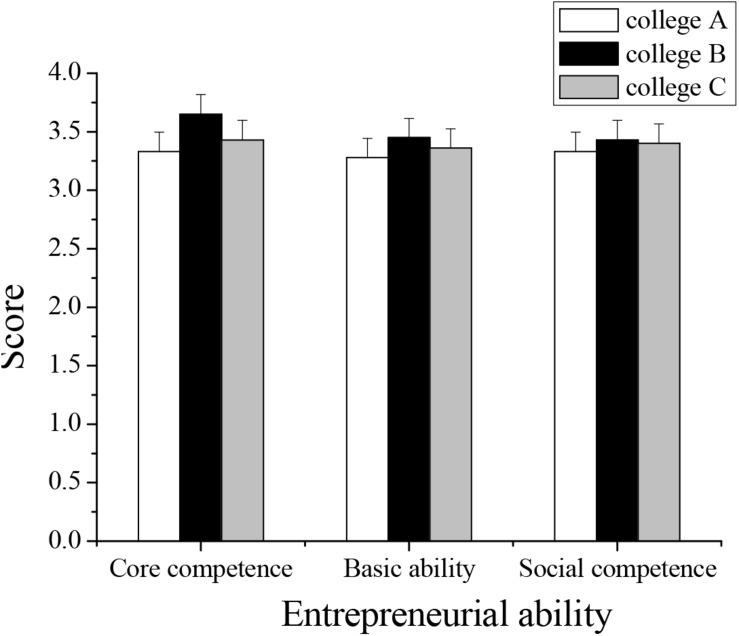
Comparison of the entrepreneurial ability of undergraduates in three universities.

4. Results of the level of entrepreneurial ability of undergraduates in three universities: Through surveys and statistics on undergraduates’ entrepreneurial ability, the core competence of the three undergraduates is (3.52 ± 0.05), the basic ability is (3.34 ± 0.03), and the social ability is (3.37 ± 0.054). The comparison of the entrepreneurial ability among the undergraduates of three universities is shown in Figure 4. As shown in Figure 4, no significant differences are found in the entrepreneurial ability of the students of the three universities. In comparison, the overall entrepreneurial ability of the students from University B is stronger, and that of the students from University A is weaker. The survey of the entrepreneurial ability of undergraduates can reflect the current states and problems of IE education in universities.

### Realization of IE Education Model in Universities and Colleges Under “Internet + Maker Education”

**FIGURE 5:**
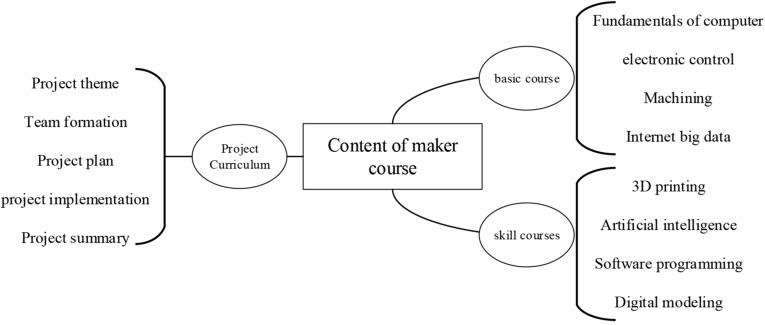
Course content of “Internet + maker education.”

**FIGURE 6:**
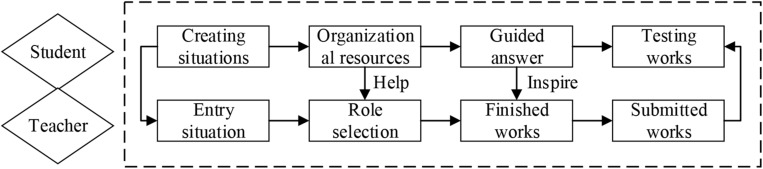
The specific learning process of undergraduates in the virtual space.

**FIGURE 7:**
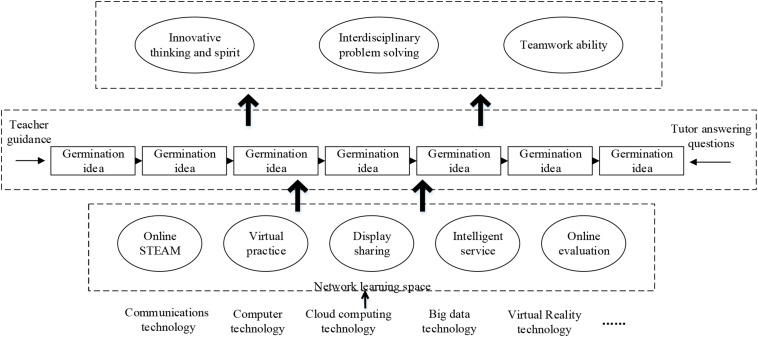
The Internet learning-based maker education model in universities and colleges.

1. The curriculum of maker education: The Internet platform provides teachers and undergraduates with a digital and intelligent learning environment. The maker course will focus on the realization of multidisciplinary knowledge and innovation. Teachers can upload courses at any time, and undergraduates can freely choose the class time. The system will automatically record the learning behaviors of each student, and the statistics are used as a reference. Undergraduates can also freely create groups to discuss the content of the course, inspiring their creative potential. The course content of “Internet + maker education” is shown in Figure 5.

2. Student learning process: Maker education on the Internet not only provides a convenient teaching environment for teachers and undergraduates, but also provides convenience for teachers and undergraduates by using various software on the Internet as auxiliary teaching tools, such as Edius, PS, and CAD. In addition, in the virtual space of the Internet, undergraduates will also have the experience brought by traditional classrooms. In the virtual classroom on the Internet, teaching scenes with sounds and pictures can also be constructed. Teachers can post tasks in the virtual space. Undergraduates can directly interact with group members through the headset, propose their innovative ideas, and use the tools to collaborate to complete the task together. Such an experience can deepen the understanding of undergraduates toward things, as well as cultivate the divergent thinking of undergraduates. The specific learning process of undergraduates in the virtual space is shown in Figure 6.

3. Maker education model: Based on the principles of practicability, interactivity, and individuality of maker education, the functional characteristics of the online learning space are combined. Then, a maker education model for universities based on Internet learning is proposed, as shown in Figure 7. This education model takes the main process of “sprouting ideas – determining the theme – online learning – online design – the creation of works – display and communication – evaluation and reflection,” which has the functional characteristics of online learning space and the characteristics of maker education. Sprouting ideas are to encourage students to think in different ways and from different perspectives. Teachers can use multimedia resources to create life-related scenes and life-like questions, which arouse students’ interests. According to their interests and creative ideas, students establish or join maker groups, use fragmented time, and select corresponding maker courses in the online learning space for viewing and learning. Finally, students utilize the knowledge acquired in the online learning space and comprehensively apply it to fully exert their imagination and creativity. The online platforms can be connected with offline 3D printing and other machines to turn the ideas of students into real objects. Students can exchange ideas with experienced makers during the creation of works, and constantly generate new ideas. Also, students can review their works through self-evaluation, netizen evaluation, and expert evaluation to continuously improve them. Under this education model, the needs of undergraduates from different majors and different grades are met. Besides, Internet teaching fully utilizes the subjective initiative of undergraduates, stimulate the desire of undergraduates to learn, and emphasize the student-centered learning methods, thereby promoting the cultivation of undergraduates’ innovative thinking.

### Perfecting and Promoting the Model of IE Education for Undergraduates

The IE education is a systematic education system in universities and colleges, and its goal is to cultivate the spirit of IE and the consciousness of university students. The spirit of IE is the cultivation of university students’ pioneering thoughts, forming a sense of initiative, tenacity, and independence. If the spirit and consciousness of university students can be formed, it will inevitably have an important impact and positive impetus on the future career development and life track of students.

1. Establishing a complete IE education system: The innovative entrepreneurship is combined with the majors of undergraduates so that they can reflect the development results of professional fields and transform professional education from theory to practice. By combining professional courses with general courses in IE education, a targeted curriculum system is created according to the characteristics of the disciplines, so that the two are seamlessly connected.
2. Constructing a diversified platform for innovation, entrepreneurship, and education: The Internet platform is fully utilized to achieve a network of resources sharing among universities, as well as the complementary advantages through open platforms, such as WeChat subscriptions, forum and Weibo. At the same time, through the education platform to gather social power, including inviting corporate leaders to teach undergraduates and providing relevant training, the interest of undergraduates in participating in innovative entrepreneurship is stimulated.
3. Enhancing the comprehensive ability of innovative entrepreneurship of undergraduates: The core of IE education of undergraduates is to use the subjective initiative of undergraduates. Only by strengthening the practical ability of undergraduates and forming the right motivation for practice can undergraduates develop a sense of innovative entrepreneurship, motivate themselves to move forward, and thus improve their comprehensive ability (Evans et al., 2018).

## Discussion

The IE education in China is mainly to meet the needs of the transformation and development of the economy and society, thereby cultivating the innovative spirit and entrepreneurial consciousness of undergraduates as the core to stimulate their creativity. In the new era, although the overall environment of IE education for undergraduates in China has changed, the awareness of IE education for undergraduates has not been fully established (Heidi et al., 2018). In the current society, innovative talents are an important strategic resource for national development. Therefore, every effort should be made to support the development of maker education.

With the sharp increase in Internet technology, its application in the field of education enables learners to communicate with each other. Meanwhile, it can serve as an open platform to promote communication between teachers and undergraduates (Basuki et al., 2018). This study first analyzes the current state of innovative entrepreneurship in China. Based on the data resources, it integrates Internet learning into the maker education system to build an IE education model for undergraduates (Wu et al., 2019a).

According to the questionnaire survey of undergraduates from three universities in Zhejiang Province, the form of innovative entrepreneurship of contemporary undergraduates is not optimistic. It is related to the support from the families of the undergraduates and the ability of universities to provide resources for innovative entrepreneurship, which are an essential factor affecting the formation of awareness of innovative entrepreneurship of undergraduates (Chen, 2019; Wu and Song, 2019). This study provides a digital and intelligent learning environment for teachers and undergraduates by applying the Internet platform. In this process, to meet the development needs of maker education, an optimization strategy is proposed for the improvement of the IE education model for undergraduates, including improving the education system, constructing an education platform, and cultivating the comprehensive ability of innovative entrepreneurship, thereby enhancing the overall entrepreneurship and innovation awareness of undergraduates (Shen and Ho, 2020).

In the implementation stage of maker education, all implementation strategies are designed for the general process of maker activities. The policy-orientation, innovation, and entrepreneurial background are the external supports for the development of maker education, which forms a systematic university maker education implementation strategy through mutual support (Wu et al., 2019b). In addition to the general functional advantages such as resource sharing and communication, the Internet and maker education carried out by colleges and universities also provide services such as online class creation and virtual experiences for teachers and students, offering more supports for the development of maker education. At the same time, the online learning space can provide university students with a virtual learning environment that is not limited by time and space (Li et al., 2019). The research results of this study are consistent with those of Deng et al. (2019) and Su et al. (2019). Based on its core value advantages, such as creative learning and experiential learning, maker education can be used as a new idea for colleges and universities to cultivate innovative and entrepreneurial capabilities in the new era (Su et al., 2019).

## Conclusion

The college IE education of the “Internet + maker education” model can reform traditional college teaching methods and promote culturing the innovative thinking and creative ability of undergraduates. While cultivating the innovative and entrepreneurial thinking of undergraduates, it is necessary to value the comprehensive training of undergraduates’ abilities, laying the foundation for the innovative entrepreneurship of undergraduates. There are deficiencies in the research process of this study that need to be improved in the subsequent study. The optimized IE education model in this study is theoretically perfect. However, it lacks data analysis to apply it to the actual teaching process, which will be the direction of the subsequent research.

## Data Availability Statement

The datasets analyzed in this article are not publicly available because they belong to an ongoing University research team project. Requests to access the datasets should be directed to the corresponding author.

## Ethics Statement

The studies involving human participants were reviewed and approved by the University Committee. The patients/participants provided their written informed consent to participate in this study.

## Author Contributions

XS did all the related research by himself including conceptualization, methodology, formal analysis, investigation, data curation, writing – original draft preparation, writing – review and editing, visualization, and has read and agreed to the published version of the manuscript.

## Conflict of Interest

The authors declare that the research was conducted in the absence of any commercial or financial relationships that could be construed as a potential conflict of interest.
